# Performance and mix ratio optimization test of building solid waste modified double liquid grouting material

**DOI:** 10.1038/s41598-026-49300-w

**Published:** 2026-04-24

**Authors:** Tong Wu, Lanjun Liu, Yanmin Chen, Jianghong Xu

**Affiliations:** School of Intelligent Construction, Shangqiu Institute of Technology, Shangqiu, 476000 China

**Keywords:** Response surface method, Red brick powder, Grouting material, Desirability function, Mixing ratio optimization, Engineering, Materials science, Mathematics and computing

## Abstract

In order to study the influence of red brick powder and water reducing agent content on the fluidity and compressive strength of construction modified solid waste double liquid grouting material (CSWMLGM), the response surface regression model was constructed, and the experimental measured values and predicted values were compared and analyzed. The CSWMLGM optimized mix ratio was obtained by combining the desirability function. The results show that the fluidity regression model has high precision and strong reliability. The regression model of compressive strength is significantly effective and can be used for the analysis of test results. The interaction between water cement ratio and red brick powder content, water cement ratio and water reducing agent content has a significant effect on fluidity. The interaction between water-cement ratio and red brick powder content, water-cement ratio and water reducing agent content, red brick powder and water reducing agent content has a significant effect on compressive strength. The model predicts that the optimal mix ratio is water-cement ratio of 1.08, red brick powder content of 23.2%, and water reducing agent content of 0.26%. The absolute value of the relative error between the predicted value and the experimental value is less than 5%. The model can reveal the mechanism of the material and provide a new scientific basis and reference for the optimization of the mix ratio of high-value double-liquid grouting materials for building solid waste.

## Introduction

Affected by complex hydrogeological conditions, water inrush disaster has become one of the common geological disasters in the process of tunnel construction and operation, which seriously restricts the safe construction and development of underground engineering. The water inrush channel is usually composed of rock mass cracks, karst fissures, fault fracture zones, and collapse columns. It has the characteristics of concealment, complexity, suddenness, and destructiveness. It is difficult to control and has high risks^1–4^. Grouting is an effective technical means of water plugging and anti-seepage in coal mine water disaster prevention and underground engineering construction^2,3^, and grouting material is the key factor to control grouting effect.

At present, in the development of new grouting materials and the research on the modification of existing grouting materials, Xiao Li et al.^5^ developed a single-liquid inert grouting material for simultaneous grouting of slurry shields through indoor and on-site ratio tests. The material has good filling, injectability and water retention, and has high early strength. After application in tunnel engineering, it effectively reduces ground subsidence. Liu et al.^6–11^ developed a new type of VCH hydrodynamic material, which can resist the erosion of water flow at a certain flow rate. Under the condition of water flow rate lower than 0.4 m/s, the material has good anti-dispersion. Under the condition of water flow rate higher than 0.4 m/s, the material loss is greatly improved. Li et al.^12^ prepared a cement-based composite grouting material (CGM) with sulphoaluminate cement clinker and steel slag powder as the main raw materials. Zhou Yaozhen^13^ developed a new bentonite-cement-based grouting material with low water separation rate, strong dynamic water erosion resistance and low environmental protection price for the hydrogeological characteristics of water-rich sandy pebble strata. Wang^14^ developed and tested the basic properties and material characteristics of polyurethane-water glass slurry and its auxiliary slurry for the problem of easy loss and difficult injection of slurry in deep water-rich fractured rock mass. Wan et al.^15^ found that the cement–water glass slurry with a ratio of 5% bentonite, 25% fly ash, 70% cement, and a slurry volume ratio of 2 had the best properties. Liu et al.^16^ studied the performance of underwater non-dispersible concrete made of flocculant or polymer, and found that it has high cohesion and good water dispersibility. Chen Yuanjiang et al.^17^ pointed out that compared with disodium hydrogen phosphate, aluminum chloride solution has a better effect on the working performance of cement–water glass slurry. The existing research on grouting materials mostly focuses on cement, water glass and industrial waste residue, and the utilization of waste red bricks with high proportion of construction solid waste is insufficient, and there is a lack of systematic mechanism research of ' dosage-performance-engineering adaptability.

The research progress of grouting materials in rheological regulation, slurry stability and strength development at home and abroad. In the cement-based slurry system, admixtures play a key role in regulating the development of rheology, stability and strength. Bu et al.^18^ prepared a hydrophobically associating water-soluble polymer, which can be used as a constant temperature rheological agent to keep the cement slurry stable at 4–90 °C. Du J et al.^19^ developed a new type of anti-leakage cementing fluid, which can effectively reinforce the formation and annulus interface and prevent oil and gas leakage. Liu et al.^20^ used silica sand to modify cement-based materials, and revealed the influence of calcium-silicon ratio and sand particle size on high temperature strength and hydration products, which provided a basis for cement slurry design under extreme conditions. Zhang et al.^21^ replaced concrete components with 0%, 5%, and 10% silicon powder, and measured the chloride ion diffusion coefficient of 28 d with Life-365 model after 5 times of repeated loading. It was found that the higher the content of silicon powder, the lower the chloride ion diffusion coefficient of concrete. Yang and Sun^22,23^ carried out long-term natural immersion and dry–wet cycle tests by placing cement mortar in 5% Na_2_SO_3_, NaCl, NaHCO_3_ and 15% compound salt solutions respectively. It was found that the mass loss rate was the largest in NaCl, and the strength corrosion resistance coefficient was the highest in Na_2_SO_3_. Through the erosion test, it was found that seawater had an early strength enhancement effect on the stone body, but with the continuous extension of the erosion age, the deterioration of the stone body gradually increased.

Domestic and foreign scholars have carried out a lot of theoretical and predictive work on the recycling of recycled building solid waste and the performance of cement-based materials. Chen et al.^24^ used the response surface function to modify the deep neural network, and established the strength prediction model of recycled brick aggregate concrete, which significantly improved the prediction accuracy and generalization ability. Chen et al.^25^ analyzed the influence of the volume content of recycled coarse and fine aggregate on the compressive strength of concrete, and put forward the corresponding strength calculation method, which provided a theoretical basis for the ratio design of recycled aggregate concrete. Aiming at the recycling of hardened cement powder, Ge et al.^26,27^ systematically studied the effect of thermal activation system on the properties, phase evolution and microstructure of hardened cement powder, and revealed its activity evolution and thermal decomposition mechanism. Ge et al.^28^ realized the efficient separation of hardened cement powder in waste concrete through high temperature heating, ball milling and other processes, which laid a foundation for the preparation of recycled cementitious materials. Ge et al.^29,30^ further prepared low-carbon alkali-activated cementitious materials by using recycled hardened cement powder and slag, and clarified the effect of free calcium oxide on the strength, phase and microstructure of the material. The above research provides important theoretical support for the mix ratio optimization and performance research of recycled red brick powder modified grouting materials in this paper. The study of Demet DEMIR SAHIN^31^ shows that mining blasting is easy to cause environmental problems such as dust, water pollution and soil degradation. The environmental impact can be effectively reduced by standardizing blasting, dust prevention and ecological restoration, which provides a basis for the development of environmentally friendly waste grouting materials in this paper. The study of Sahin^32^ shows that the pozzolanic activity of fly ash can be significantly improved by reasonable grinding, which provides a theoretical basis for the study of fly ash composite grouting materials. Liu W K^33^ confirmed that fly ash can effectively improve the sulfate resistance of the matrix through pozzolanic reaction, which provides support for the long-term durability analysis of grouting materials. Hasan E^34^ studied the effect of material fineness on strength and chemical resistance. Demet Demir Šahin^35^ research shows that grouting and sealing operations, as important supporting links in mining, although not separately costed, fit the research objectives of cost optimization and operational efficiency improvement, and will be combined with its cost model to refine the cost accounting of such operations. Demir Sahin, D^36^ constructed an ANN model with a prediction accuracy of 99%, and used fly ash fineness, substitution rate and age as parameters to achieve effective prediction of compressive strength, which can provide support for the comparative analysis of the regression model and RSM method in this paper and improve the scientific nature of the research.

In recent years, China has vigorously carried out urban construction and old city reconstruction, which will inevitably produce a large amount of construction waste every year. Construction waste mainly includes waste concrete and waste bricks, of which waste bricks account for 35–45%^37–39^. As a kind of building solid waste material, red brick powder has the characteristics of easy access, low cost, energy saving and environmental protection. In this paper, the author takes water-cement ratio, recycled red brick powder and superplasticizer dosage as factors, and takes the fluidity and compressive strength of CSWMLGM as the response value. On the basis of orthogonal test, the response surface model is established by RSM, and the influence of various factors on the response value is analyzed. The optimal ratio of CSWMLGM is obtained by combining the desirability function, in order to provide reference for the multi-objective optimization of the mix ratio of construction solid waste modified double liquid grouting material.

## Test overview

### Materials

The ordinary Portland cement (OPC.42.5) produced by Hubei Huaxin Cement Co., Ltd.is selected as the main grouting material, and the chemical composition is shown in Table1. The accelerator used in the test is liquid water glass, which is purchased from Sinopharm Chemical Reagent Beijing Co., Ltd., and conforms to the current national standard Technical specification for cement–water glass double liquid grouting in construction engineering JGJ/ T211-2010. The main parameters are shown in Table 2. The waste red brick used in is taken from the construction waste generated by urban construction and old city reconstruction. After a certain degree of crushing, the red brick powder is obtained. Its fineness is 200–250 mesh, and the main chemical composition is shown in Table 3. Red brick powder and fly ash have many similar characteristics. The detailed comparison of their properties is shown in Table 4.

**Table 1:** Chemical composition of ordinary silicates.

Type	SiO_2_	Fe_2_O_3_	Al_2_O_3_	CaO	MgO	K_2_O
OPC	22.34	2.76	6.03	56.98	1.86	0.73

**Table 2:** Main technical parameters of water glass.

Appearance	Baume degres (Bé)	Density (g/cm^3^)	Na_2_O (%)	SiO_2_ (%)	Modulus
Slightly yellow viscous liquid	39	1.373	8.35	27.41	3.21

**Table 3:** Chemical composition of red brick powder.

Type	SiO_2_	TiO_2_	Al_2_O_3_	MnO	MgO	CaO	Na_2_O	K_2_O	LOI
Red brick powder	62.89	0.72	14.21	5.33	2.58	8.17	1.77	2.68	2.05

**Table 4:** Material properties of red brick powder and volcanic ash.

Characteristic index	Testing method	Characteristics of red brick powder	Volcanic ash activity characteristics	Data sources
Chemical Composition (XRF)	SiO_2_: 50–65%, Al_2_O_3_: 15–25%, CaO: 3–8%, Fe_2_O_3_: 4–7%, the rest is a small amount of MgO, K_2_O	SiO_2_, Al_2_O_3_ are the main active components, and the content of CaO is low, so it is difficult to stimulate the activity	SiO_2_, Al_2_O_3_ are the main active components, and the content of CaO is low, so it is difficult to stimulate the activity	^37–39^
Phase composition (XRD)	Main phase is quartz, mullite, containing a small amount of hematite, calcite, no obvious active mineral phase	The crystal structure is stable, and the degree of secondary hydration reaction with cement hydration product Ca (OH)_2_ is low	The crystal structure is stable, and the degree of secondary hydration reaction with cement hydration product Ca (OH)_2_ is low	^38,39^
Microstructure (SEM)	Particles are irregularly angular with compact surface and low porosity. The specific surface area is 180–220 m^2^/kg	There are few surface active sites, and the interface bonding with the hydrated gel is mainly physical adsorption	There are few surface active sites, and the interface bonding with the hydrated gel is mainly physical adsorption	^37,39^
Pozzolanic activity index	The 28d activity index was 35–45%	It belongs to low pozzolanic active material, and the secondary hydration reaction is weak	It belongs to low pozzolanic active material, and the secondary hydration reaction is weak	^37,38^

### Test method

According to DL/T5150-2017 Hydraulic Concrete Test Regulations, the composite grouting material was prepared, and the fluidity and compressive strength tests were selected as indicators to evaluate the performance of grouting materials.The test adopts a two-liquid grouting system : liquid A is cement-red brick powder-water reducing agent slurry, and liquid B is water glass solution. Firstly, cement, red brick powder and water reducing agent were mixed evenly according to the design mix ratio. After adding quantitative water, liquid A was prepared by stirring at low speed of 140r/min for 120 s in JJ-5 cement mortar mixer. Liquid A and liquid B were then rapidly mixed at a volume ratio of 1:1.

#### Fluidity test

The fluidity of CSWMLGM slurry was characterized by micro-slump test. The test mold was used on smooth glass^40^. The mold size was 60 mm high, 36 mm bottom diameter and 60 mm top diameter. The mold filled with slurry is quickly lifted from the glass plate, and then the slurry expands freely on the glass plate for 30 s. The maximum diameter of the slurry in the transverse and longitudinal directions is measured, and the average value is taken. This value is the slurry fluidity

#### Compressive strength test

In this test, the performance test of uniaxial compressive strength of specimen was carried out according to the relevant provisions of JGJ/T70-2009" Standard for Basic Performance Test Method of Building Mortar. In this experiment, a 50 mm × 100 mm cylinder mold was used for molding. After standing for 24 h in a standard environment with a temperature of (20 ± 2)°C and a relative humidity of ≥ 95%, the mold was demoulded, and the standard curing was continued to 28 d. The compressive strength was measured according to the specification. The loading rate is adjusted according to the estimated mortar strength by continuous uniform loading: when the slurry strength is less than or equal to 5 MPa, the loading rate is 0.2–0.5kN/s; when the slurry strength is greater than 5 MPa, the loading rate is 1.0–1.5kN/s.

In this study, fluidity and 28d compressive strength are the core evaluation indexes: fluidity directly determines the groutability and construction performance of grout, and 28 d compressive strength reflects the long-term bearing capacity of stone body. The two are the most basic and representative performance indexes of grouting materials, which can effectively reveal the influence of water-cement ratio, red brick powder content and water reducing agent content on materials. The engineering application indexes such as setting time, bleeding rate, stone rate and water dispersion resistance will be supplemented in the subsequent system test.

### Experimental design

In order to standardize the scientific design of building mortar mix ratio, the CSWMLGM mix ratio design is carried out with reference to the ' ordinary building mortar mix ratio design specification '. Water cement ratio (factor A) design 0.8, 1.0 and 1.2; the proportion of red brick powder (factor B) is 20%, 40% and 60%, Its dosage is calculated according to the same quality instead of cement, and the calculation method is shown in the formula (1). The content of water reducing agent (factor C) is 0.25%, 0.5% and 0.75%. For these three factors, the test data were processed using the BBD design function in Design − expert 10.0.7 software, the application of BBD in the field of concrete and mortar is shown in Table 5. Based on the existing research and considering the feasibility of trial matching, the specific range of each level is determined. The experimental factors and levels are shown in Table 6. According to the test conditions in Table 6, a total of 13 groups of different mix proportions were designed.

1 $$Cotent(B) = \frac{quality(B)}{{quality(A + B + C)}} \times 100{{\% }}$$

**Table 5:** Application comparison of RSM-BBD method in the field of cementitious materials.

Authors	Research system	Influence factors	Optimization results
Sun et al.^40^	Iron tailings modified reactive powder concrete	Water cement ratio, mineral powder content, water reducing agent content	Under the optimal ratio, the compressive strength increases by 12.5%, and the fluidity meets the engineering requirements
zhu et al.^41^	Fly ash modified cement mortar	Fly ash content, sand cement ratio, water cement ratio	Model R^2^ > 0.95, the relative error between the predicted value and the experimental value is less than 4%
Ba et al.^42^	Steel slag powder modified cement-based grouting material	Steel slag content, water cement ratio, accelerator content	Initial setting time of the optimal ratio is shortened by 28%, and the 28 d compressive strength is increased by 9.8%

**Table 6:** Experimental factors and levels.

Level	Factor		
	A	B/%	C/%
-1	0.8	20	0.25
0	1	40	0.5
1	1.2	60	0.75

In the formula: B is the mass of red brick powder (g); a is the quality of cement (g); c is the quality of water reducing agent (g).

## Results and analysis

### Test results

The experimental and predicted values of fluidity and 28d compressive strength of CSWMLGM are shown in Table 7.

**Table 7:** Test design and results.

Event number	A	B/%	C/%	Fluidity (cm)		28d Compressive strength (MPa)	
				Measured value	Predicted value	Measured value	Predicted value
1	1.2	40	0.75	32.8	32.85	5.5	5.62
2	1.2	60	0.5	33.2	33.22	6.5	6.40
3	1	20	0.75	27.4	27.37	6.1	5.49
4	1	60	0.25	28.4	28.42	6.9	7.84
5	0.8	40	0.75	26.2	26.25	9.3	9.62
6	1	60	0.75	31.8	31.72	10.2	9.76
7	0.8	20	0.5	22.8	22.77	6.7	6.57
8	1	40	0.5	25.2	25.2	7	8.61
9	1.2	40	0.25	28.9	28.85	12.3	11.05
10	1	20	0.25	23.8	23.87	10.6	11.36
11	1.2	20	0.5	27.3	27.27	10.2	10.27
12	0.8	40	0.25	23.5	23.45	9.2	9.76
13	0.8	60	0.5	25.7	25.72	11.5	11.2

### Response surface model establishment and variance analysis

The Design-expert software was used to perform multiple regression fitting analysis on the measured values in Table 7, and the quadratic polynomial response surface regression equations of fluidity (Y_1_), compressive strength (Y_2_) and independent variable factors A, B, and C were established:

2 $$\begin{aligned} {\mathrm{Y}}_{{1}} = & {25}.{2} + {3}.0{\mathrm{4A}} + {2}.{\mathrm{26B}} + {1}.{\mathrm{7C}} + 0.{\mathrm{825AB}} + 0.{\mathrm{3AC}} - 0.0{\mathrm{5BC}} \\ & + {1}.0{\mathrm{6A}}^{{2}} + {1}.0{\mathrm{6B}}^{{2}} + {1}.{\mathrm{59C}}^{{2}} \\ \end{aligned}$$

3 $${\mathrm{Y}}_{{2}} = {8}.{62} - 0.{\mathrm{275A}} + 0.{\mathrm{1875B}} - 0.{\mathrm{9875C}} - {2}.{\mathrm{12AB}} - {1}.{\mathrm{73AC}} + {1}.{\mathrm{95BC}}$$

Variance analysis and reliability analysis were performed on the above regression models, and the results are shown in Tables 8, 9, 10. In this paper, P value is used to evaluate the significance of the model. The P value represents the probability that the initial hypothesis is true. The larger the F value, the smaller the P value, indicating that the model is more significant^40^. When the *P* value > 0.05, the significance of the model is low, and the regression model is not available; on the contrary, the regression model is more significant^43^. Correlation coefficient R_2_, adjustment coefficient R_a_^2^ coefficient of variation and signal-to-noise ratio were used to evaluate the reliability of the model. The correlation coefficient R_2_ represents the degree of difference between the response value and the true value, and the value range is 0–1. The larger the value, the smaller the difference between the two. The smaller the difference between the adjustment coefficient R^2^_a_ and the correlation coefficient R_2_, the smaller the coefficient of variation, and the signal-to-noise ratio > 4, indicating that the higher the fitting degree of the regression equation, the higher the reliability and accuracy of the test.

**Table 8:** Variance analysis of mobility regression model.

Data source	Quadratic sum	Degree of freedom	Mean square	F-number	*p*-value	Significant or not
Y_1_	143.85	9	15.98	1917.94	< 0.0001	Yes
A	72.00	1	72.00	8640.00	< 0.0001	Yes
B	39.60	1	39.60	4752.60	< 0.0001	Yes
C	23.12	1	23.12	2774.40	< 0.0001	Yes
AB	2.25	1	2.25	270.00	0.0005	Yes
AC	36	1	0.3600	43.20	0.0072	Yes
BC	0.01	1	0.0100	1.20	0.3534	No
A^2^	2.4	1	2.40	288.17	0.0004	Yes
B^2^	2.4	1	2.40	288.17	0.0004	Yes
C^2^	6.04	1	6.04	724.29	0.0001	Yes
Residual	0.0250	3	0.0083			
Cor Total	143.87	12

**Table 9:** Analysis of variance of regression model for compressive strength.

Data source	Quadratic sum	Degree of freedom	Mean square	F-number	P-value	Significant or not
Y_2_	53.86	6	8.98	7.15	0.0152	Yes
A	0.6050	1	0.6050	0.4818	0.5136	No
B	0.2813	1	0.2813	0.2240	0.6528	No
C	7.80	1	7.80	6.21	0.0470	Yes
AB	18.06	1	18.06	14.38	0.0090	Yes
AC	11.90	1	11.90	9.48	0.0217	Yes
BC	15.21	1	15.21	12.11	0.0131	Yes
Residual	7.53	6	1.26			
Cor Total	61.40	12

**Table 10:** Model credibility testing.

Model	Std.Dev	Mean	*R*^*2*^	Adjusted *R*_*a*_^*2*^	Adeq precision	C.V.%
Y_1_	0.0913	27.46	0.9998	0.9993	130.52	0.332
Y_2_	1.12	8.62	0.8773	0.7546	7.1447	13.01

It can be seen from Tables 8 and 10 that the Y_1_ regression model has high significance and reliability. The P value of the regression model Y_1_ was < 0.0001, and the F value was 1917.94, indicating that the significance of the regression model was high. At the same time, the correlation coefficient R_2_ is 0.998, the adjustment coefficient R^2^_a_ is 0.999, the coefficient of variation is 0.332%, and the signal-to-noise ratio is 130.52, indicating that the regression model has high reliability. The P values of AB and AC in Y_1_ were < 0.05, the P values of BC were > 0.05, and the P values of A, B and C were < 0.05, indicating that the interaction of two-factor AB and AC had a significant effect on the fluidity, and the interaction of BC had a small effect on the fluidity, while the content of single factor A, B and C also had a significant effect on the fluidity.

It can be seen from Tables 9 and 10 that the R^2^ and adjusted Radj^2^ of the fluidity model Y_1_ are close to 1, the coefficient of variation is extremely low, the signal-to-noise ratio is much larger than 4, and the model has high accuracy and strong credibility. The fitting effect of the compressive strength model Y_2_ is medium, which is significantly lower than that of Y_1_, but the model is significant as a whole, which can be used for mix proportion optimization analysis and rule prediction. In addition, the P value of C, AB, AC and BC in Y_2_ was 0.05, indicating that the interaction of single factor C, double factor AB, AC and BC had a significant effect on compressive strength, while single factor A and B had little effect on compressive strength.

**Fig. 1:**
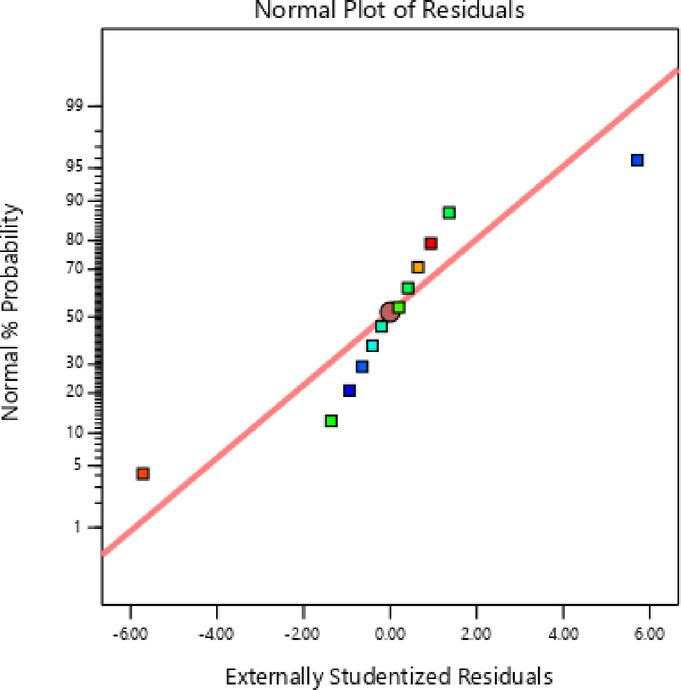
Student residual of mobility reduction compressive strength model.

**Fig. 2:**
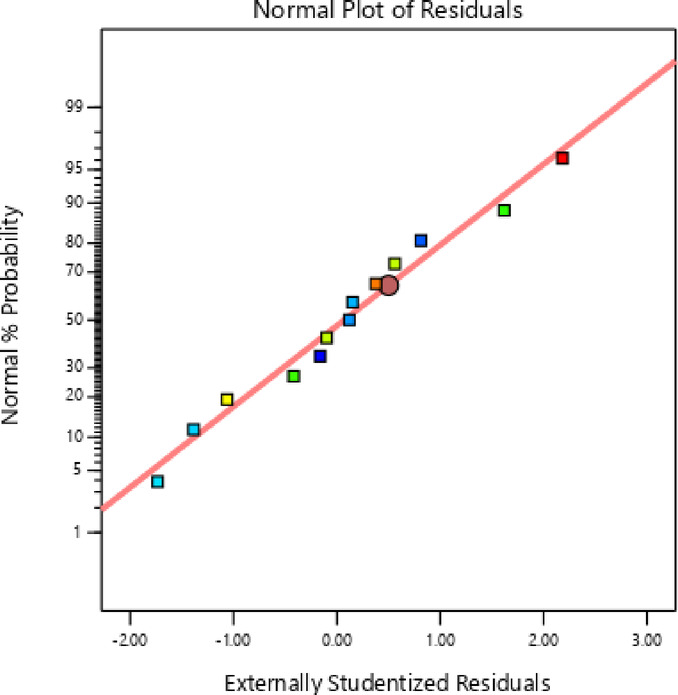
Student residual of regression model.

The studized residuals of regression models can be derived by using the ANOVA function of Design-Expert software. The studized residuals play a very important role in the diagnosis of regression models^44^. The value obtained by dividing the finger residuals by the standard deviation can intuitively determine whether the residuals follow the normal distribution. The studized residuals of the regression models for mobility and compressive strength are shown in Figs. 1 and 2 respectively. As can be seen from the analysis diagram, there are no abnormal data points in the regression models, and the data points are evenly distributed near the straight lines, indicating that the residual error is small and the fitting effect of the model is ideal.

### Response surface analysis

**Fig. 3:**
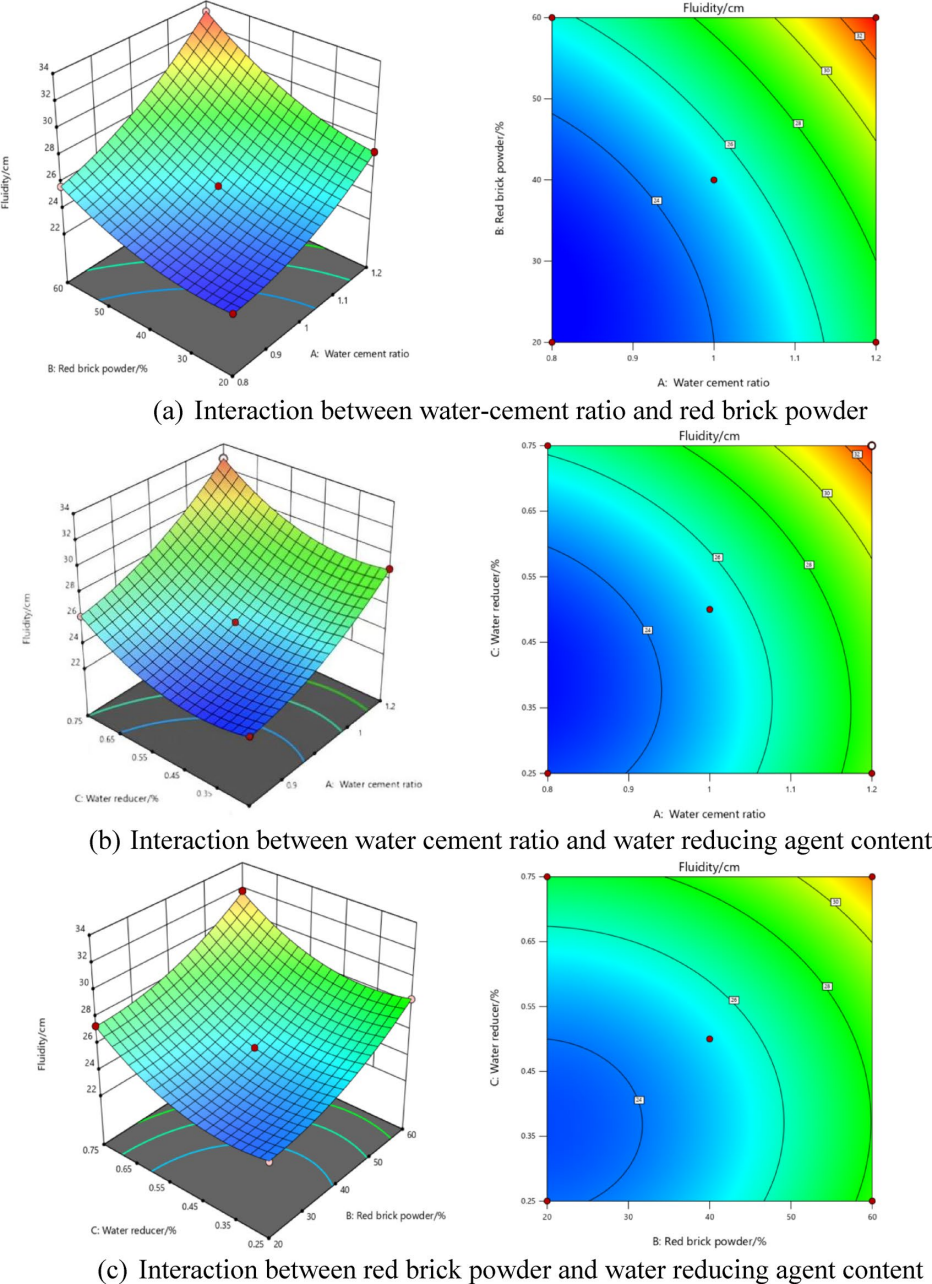
Flow response surface diagram and contour diagram.

**Fig. 4:**
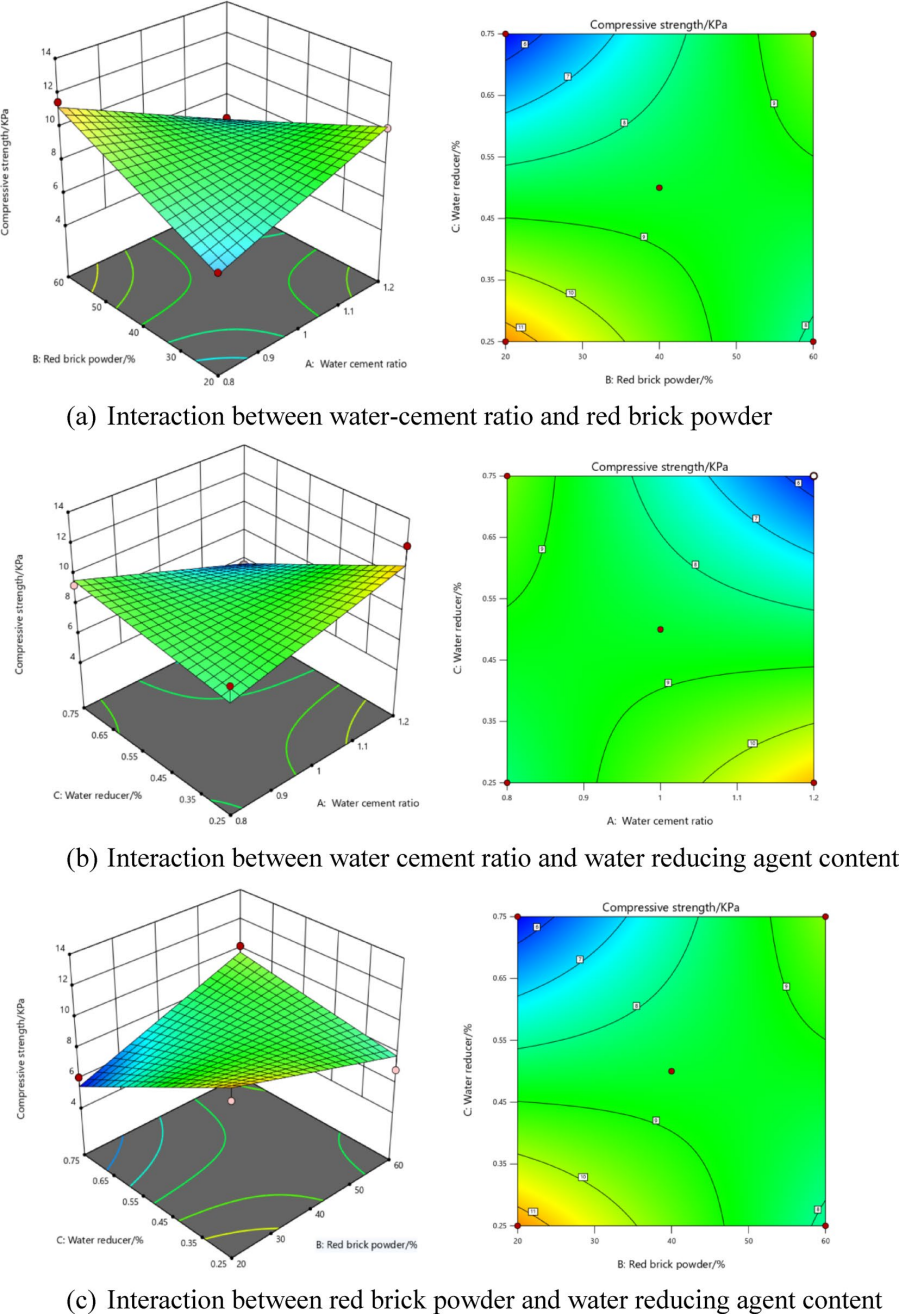
Response surface plots and contour diagram of compressive strength.

According to the linear regression model, the 3D response surface diagram and the corresponding contour map of the interaction of the two factors were drawn respectively, which can be used to intuitively analyze the influence of the interaction of the two factors on the fluidity and compressive strength of CSWMLGM. The results are shown in Figs. 3 and 4. When the contour shape is elliptical, it indicates that the interaction between the two factors is significant; the circle indicates that the interaction is not significant. The denser the intersection of the contour line and the coordinate, the greater the influence of this factor on the response value.

#### Fluidity analysis

It can be seen from Fig. 3a that when the amount of water reducing agent is 0.5% and the water-cement ratio is greater than 0.8, the fluidity increases with the increase of water-cement ratio. When the content of red brick powder is more than 40%, the fluidity increases with the increase of red brick powder content. When the content of red brick powder is not more than 40%, the fluidity has no obvious change with the increase of red brick powder content. The fluidity has a maximum value under the appropriate water-cement ratio and red brick powder content. The maximum value appears when the water-cement ratio is in the range of 1–1.2 and the red brick powder content is in the range of 40–60%.

It can be seen from Fig. 3b that the interaction between different water-cement ratios and water reducing agent content is more significant. And the water-cement ratio has a greater impact on the fluidity. When the content of red brick powder is 40%, when the content of water reducing agent exceeds 0.5%, the fluidity increases with the increase of water reducing agent content. When the dosage of water reducing agent is less than 0.5%, the fluidity has no obvious change with the increase of the dosage of water reducing agent.

From Fig. 3c, it can be seen that when the water-cement ratio is 1 and the red brick powder content is greater than 20%, the fluidity increases with the increase of the red brick powder content; when the dosage of water reducing agent is less than or equal to 0.5%, the fluidity has no obvious change with the increase of the dosage of water reducing agent. When the substitution rate of RBA is higher than 0.5%, the fluidity has no obvious change with the increase of water reducing agent content.

#### Compressive strength analysis

It can be seen from Fig. 4a that when the amount of fixed water reducing agent is 0.5%, the amount of red brick powder increases and the compressive strength gradually increases in the range of water-cement ratio of 0.8–1. In the range of water cement ratio 1–1.2, the content of red brick powder continues to increase, and the compressive strength shows a downward trend. On the whole, an appropriate amount of red brick powder under low water-cement ratio can play a role in filling enhancement, and too much red brick powder under high water-cement ratio will reduce the compactness of the system, resulting in a decrease in strength. It can be seen from Fig. 4b that when the content of fixed red brick powder is 40%, the water-cement ratio is in the range of 0.8–1, and the compressive strength increases with the increase of water-cement ratio. When the water-cement ratio exceeds 1, the compressive strength decreases with the increase of water-cement ratio. The increase of water reducing agent content will significantly improve the dispersion and compactness of the slurry, so that the compressive strength increases first and then decreases, and there is an optimal dosage range. It can be seen from Fig. 4c that when the fixed water-cement ratio is 1, the compressive strength increases slightly with the increase of water reducing agent when the content of red brick powder is in the range of 20–40%. When the content of red brick powder exceeds 40%, the increase of water reducing agent content weakens the effect of strength improvement. The water reducing agent can improve the dispersion state of the particles, but excessive amount will introduce too many bubbles, resulting in a decrease in strength.

The red brick powder mainly improves the compactness of the stone body by physical filling. When the content of red brick powder is 20–40%, the red brick powder fills the gap of cement particles, the structure is denser and the compressive strength is improved. When the content of is more than 40%, the amount of cement is insufficient, the bonding performance of the slurry decreases, and the strength decreases. When the amount of water reducing agent is appropriate, the particles can be significantly dispersed and the agglomeration can be reduced. If the content is too high, it is easy to introduce pores and weaken the structural compactness. When the content of water reducer is less than 0.5%, the increase of red brick powder content leads to the increase of friction between particles, while the dispersion effect of water reducer is insufficient, and the compressive strength decreases. When the dosage of water reducing agent is higher than 0.5%, the water reducing agent can effectively disperse the cement and red brick powder particles, alleviate the agglomeration problem under high dosage, improve the interface bonding quality, and increase the compressive strength.

### Multi-objective optimization and verification of the model

Combined with the research results of similar dynamic water grouting by Liu et al.^6,11^, Zhou Yao^13^ and Wan Zhi et al.^15^, in the treatment of water gushing in tunnels with medium and low flow rates of 0–0.4 m/s, the cement–water glass double liquid grouting material needs to meet the engineering requirements of initial setting time of 5–15 min, final setting time of 20–30 min and water separation rate of ≤ 3%, which also defines a clear performance reference standard for the subsequent engineering application of this material. However, the single-objective optimization is difficult to take into account the requirements of fluidity, compressive strength and the key performance indicators of the above projects at the same time. Therefore, it is urgent to achieve the optimal matching of the comprehensive performance of grouting materials through multi-objective optimization. Based on the response surface regression model and combined with the numerical optimization function of the desire function in Design-Expert software, the multi-objective optimization of CSWMLGM was carried out under the conditions of different water-cement ratio (factor A), red brick powder content (factor B) and water reducing agent (factor C). The CSWMLGM fluidity regression model (Y_1_) meets the maximum value of the two-liquid grouting technical specification and the compressive strength regression model (Y_2_). The desire function (D) is defined as the product of the power exponent of each response desire value, and the desire value (*d*_*i*_) of a single response value is within the range of 0–1. The closer D is to 1, the more reliable the optimal condition will be obtained. The expressions of the desire value (*d*_*i*_) and the desire function (D) are as follows:

4 $$d_{\max } = \left\{ {\begin{array}{*{20}l} {0,} \hfill & {y \le L} \hfill \\ {\left[ {\frac{y - L}{{U - L}}} \right]^{{w_{i} }} ,} \hfill & {L \le y \le U} \hfill \\ {1,} \hfill & {y > U} \hfill \\ \end{array} } \right.$$

5 $$d_{\min } = \left\{ {\begin{array}{*{20}l} {1,} \hfill & {y \le L} \hfill \\ {\left[ {\frac{U - y}{{U - L}}} \right]^{{w_{i} }} ,} \hfill & {L \le y \le U} \hfill \\ {0,} \hfill & {y > U} \hfill \\ \end{array} } \right.$$

6 $$D = d_{1}^{{w_{1} }} d_{2}^{{w_{2} }}$$

In the formula:* y* is the fitting value of the *i* response; *L* is the lower limit value of the *i* response, and *U* is the upper limit value of the *i* response surface. *w*_*i*_ is the weight of the *i* response.

The optimization design objectives are shown in Table 11. The optimal Design objectives of CSWMLGM mix ratio optimization and the value range of optimization parameters in Table 8 were input into Design-Expert software to obtain the optimal design results of the optimal fit ratio and response surface design, as shown in Table 12.

**Table 11:** Optimization design objectives of CSWMLGM.

Parameters to be optimized	Value ranges		Required value
	Minimum value	Maximum value	
A	0.8	1.2	0.8–1.2
B/%	20	60	20–60
C/%	0.25	0.75	0.25–0.75
Y_1_/cm	22.8	33.2	10–30
Y_2_/MPa	5.5	12.3	Value ranges

**Table 12:** Optimal design parameters of response surface design.

Water-cement ratio	Red brick powder/ (%)	Water reducing agent/ (%)	Fluidity/cm	Compressive strength/MPa	Desire function value
1.08	23.2	0.26	24.9	12.3	0.83

In order to further verify the accuracy of the model, the optimal mix ratio shown in Table 9 is tested and verified, and the experimental value is compared with the predicted value. The results are shown in Table 13, and the absolute value of the relative error is calculated by the calculation formula (7). Among them, E_1_ and E_2_ are the absolute values of relative errors of fluidity and compressive strength respectively. It can be seen from Table 10 that under the optimal mix ratio, E_1_ and E_2_ are less than 5%, indicating that the regression model of fluidity and compressive strength has high prediction accuracy^25^. Therefore, the optimization of CSWMLGM mixture ratio by RSM can improve the test efficiency and obtain the suitable slurry ratio parameters for water gushing treatment.

7 $$E = \frac{{\left| {V_{T} - V_{P} } \right|}}{{V_{T} }} \times 100\%$$

**Table 13:** Verification of optimal design parameters.

Y_1_/cm		E_1_/%	Y_2_/MPa		E_2_/%
Predicted value	Experimental value		Predicted value	Experimental value	
24.9	24.2	2.8	12.32	11.8	4.2

In the formula:* E* is the absolute value of relative error;*V*_*T*_ fluidity or compressive strength test ; *V*_*P*_ is the predicted value of fluidity or compressive strength.

## Conclusion

1. The regression model of CSWMLGM fluidity and compressive strength has high accuracy and credibility, which can be used to simulate and analyze the test results.
2. For the fluidity of CSWMLGM, the water-cement ratio, red brick powder and water reducing agent content have a significant effect on it ; the compressive strength of CSWMLGM was significantly affected by water reducer agent and all two-factor interactions.
3. For the fluidity of CSWMLGM, the interaction of two factors AB and AC had a significant effect on the fluidity, and the interaction of BC had little effect on the fluidity. For the compressive strength of CSWMLGM, the interaction of two-factor AB, AC and BC has a significant effect on the compressive strength.
4. The optimal water-cement ratio predicted by the model is 1.08, the content of red brick powder is 23.2%, and the content of water reducing agent is 0.26%. The absolute value of the relative error between the predicted value and the experimental value is less than 5%, indicating that the model can provide a reference for the multi-objective optimization of CSWMLGM mix ratio.
5. This study mainly completed the mix ratio optimization and mechanism analysis of red brick powder modified grouting materials. In the future, key engineering indicators such as setting time, bleeding rate, water separation rate, stone rate, viscosity time-varying characteristics, anti-dispersion and early strength will be further tested to improve the material system and enhance engineering applicability.

## Data Availability

The datasets used and/or analyzed during the current study are available from the corresponding author on reasonable request.
